# Reconstructing the evolutionary history of the radiation of the land snail genus *Xerocrassa *on Crete based on mitochondrial sequences and AFLP markers

**DOI:** 10.1186/1471-2148-10-299

**Published:** 2010-10-04

**Authors:** Jan Sauer, Bernhard Hausdorf

**Affiliations:** 1 Zoological Museum, University of Hamburg, Martin-Luther-King-Platz 3, 20146 Hamburg, Germany; 2 Department of Chemical Ecology, University of Bielefeld, Universitätsstraße 25, 33615 Bielefeld, Germany

## Abstract

**Background:**

A non-adaptive radiation triggered by sexual selection resulted in ten endemic land snail species of the genus *Xerocrassa *on Crete. Only five of these species and a more widespread species are monophyletic in a mitochondrial gene tree. The reconstruction of the evolutionary history of such closely related species can be complicated by incomplete lineage sorting, introgression or inadequate taxonomy. To distinguish between the reasons for the nonmonophyly of several species in the mitochondrial gene tree we analysed nuclear AFLP markers.

**Results:**

Whereas six of the eleven morphologically delimited *Xerocrassa *species from Crete are monophyletic in the mitochondrial gene tree, nine of these species are monophyletic in the tree based on AFLP markers. Only two morphologically delimited species could not be distinguished with the multilocus data and might have diverged very recently or might represent extreme forms of a single species. The nonmonophyly of *X. rhithymna *with respect to *X. kydonia *is probably the result of incomplete lineage sorting, because there is no evidence for admixture in the AFLP data and the mitochondrial haplotype groups of these species coalesce deeply. The same is true for the main haplotype groups of *X. mesostena*. The nonmonophyly of *X. franciscoi *might be the result of mitochondrial introgression, because the coalescences of the haplotypes of this species with some *X. mesostena *haplotypes are shallow and there is admixture with neighbouring *X. mesostena*.

**Conclusion:**

The most likely causes for the nonmonophyly of species in the mitochondrial gene tree of the *Xerocrassa *radiation on Crete could be inferred using AFLP data by a combination of several criteria, namely the depth of the coalescences in the gene tree, the geographical distribution of shared genetic markers, and concordance with results of admixture analyses of nuclear multilocus markers. The strongly subdivided population structure increases the effective population size of land snail species and, thus, the likelihood of a long persistence of ancestral polymorphisms. Our study suggests that ancestral polymorphisms are a frequent cause for nonmonophyly of species with a strongly subdivided population structure in gene trees.

## Background

The delimitation of closely related species and the reconstruction of their evolutionary history can be complicated by shared ancestral polymorphisms, introgression or inadequate taxonomy [1]. These problems may result in discrepancies between gene trees and a species classification based on other data, which may became apparent if the markers are sequenced from several individuals of each species. The probability that ancestral polymorphisms are shared between species increases with decreased time between speciation events [2-6]. The likelihood that isolation mechanisms are incomplete and introgression happens is also higher if species originated in a short time span and, hence, were at least initially genetically similar [1,7,8]. Inadequate taxonomy, the third cause for discrepancies between gene trees and a species classification, is more likely, if several species originated in a short period of time and are morphologically similar. Thus, the delimitation of species and the reconstruction of their relationships are especially challenging in species radiations [9]. Species that are nonmonophyletic in gene trees have been found in diverse groups, e.g., in radiations of East African cichlids [10-12], Darwin's finches [13], Moorean tree snails [14], Hawaiian swordtail crickets [15], North American lycaenid butterflies [16], ambystomatid salamanders [17], Argentinean liolaemid lizards [18], North American crotaphytid lizards [19], and barley [20].

**Figure 1:**
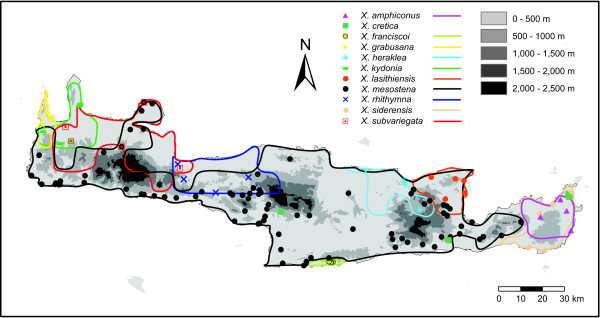
**Distribution of the endemic *Xerocrassa *species on Crete**. Symbols indicate sampling sites of the *Xerocrassa *specimens for which AFLP data were determined.

We investigated the radiation of the land snail genus *Xerocrassa *(Gastropoda: Helicoidea: Hygromiidae) on Crete. Eleven native *Xerocrassa *species can be distinguished on Crete based on morphological characters of the shell and the genitalia [21]. All native species living on Crete are endemic (Figure 1) except the more widespread *Xerocrassa cretica*. It has been supposed that the radiation of *Xerocrassa *on Crete was a non-adaptive radiation probably triggered by sexual selection [22]. Only six of the morphologically defined species are monophyletic in a mitochondrial gene tree [22]. We checked the morphological species delimitation using a tree and a network based on AFLP markers [23] and tested the efficiency of several approaches for delimiting genotypic clusters based on multilocus data without a prior knowledge of the number of species. We used an integrative approach combining several criteria to discriminate between incomplete lineage sorting and introgression as causes for the nonmonophyly of species in the mitochondrial gene tree of the *Xerocrassa *radiation on Crete.

## Results

### Mitochondrial gene tree

**Figure 2:**
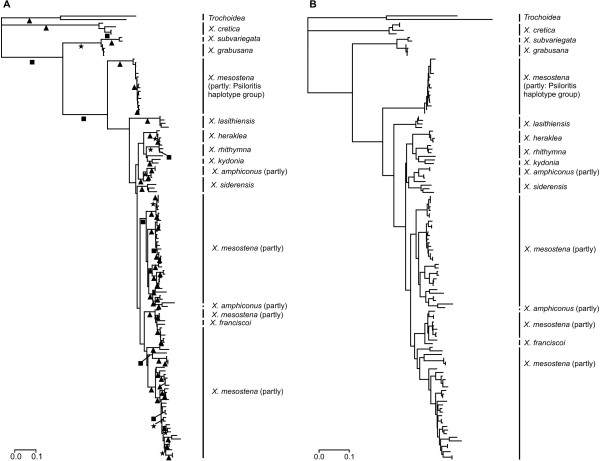
**Maximum likelihood trees of partial COI sequences of 122 Cretan *Xerocrassa *and two *Trochoidea *specimens**. (A) Maximum likelihood tree calculated with a stationary model. Bootstrap support is indicated by symbols below the branches (stars = 70-80%, squares = 80-90%, triangles = 90-100%). (B) Maximum likelihood tree calculated with a nonstationary model.

Separate models for the three codon positions as determined by ModelTest based on the AIC were used for the maximum likelihood analysis (1. codon positions: TrN+I+G, 2. codon positions TrN+I, 3. codon positions TVM+G), because the resulting tree had a lower AIC value (-lnL = 9246.20; AIC = 18986.39) than the tree based on a uniform model for the complete dataset (TVM+I+G; -lnL = 9638.11; AIC = 19766.21). The maximum likelihood tree of 122 partial COI sequences (634 bps) of Cretan *Xerocrassa *species and two *Trochoidea *species as outgroups is shown in Figure 2A (see also [21]). COI GTR+G distances varied from 0.8% to 33.0% (mean 18.7%) between Cretan *Xerocrassa *species and from 0.0% to 25.0% (mean 13.6%) within Cretan *Xerocrassa *species.

According to chi-square tests the base composition at the first and second codon positions of the used COI sequences are not heterogeneous (*p *= 1.000), but there is significant heterogeneity at the third codon positions (*p *= 0.016). The results of the matched-pairs tests of symmetry are compatible with these results. According to the matched-pairs tests of symmetry 37.5% of the pairwise comparisons of the nucleotides at the third codon positions indicate significant (*p *< 0.050) heterogeneity, whereas only 4.0% of the pairwise comparisons of the nucleotides at the first codon positions and 0.0% of the pairwise comparisons of the nucleotides at the second codon positions indicate significant heterogeneity.

To reduce the compositional heterogeneity at the third codon positions we recoded the nucleotides at the third codon positions as purines and pyrimidines. This RY-recoding resulted in a loss of information so that the resulting tree was poorly supported. The haplotype group of *X. mesostena *that is sister to all other haplotypes of endemic species except *X. subvariegata *and *X. grabusana *(Figure 2) became nested in *X. siderensis *haplotypes from eastern Crete. This haplotype group of *X. mesostena *is distributed in a region southwest of the Psiloritis Mountains and will be called the 'Psiloritis haplotype group' in the following.

The analysis with the nonstationary model implemented in nhPhyML-Discrete requires a starting tree. We used the maximum likelihood tree obtained with the unmodified dataset as well as the maximum likelihood tree obtained with the RY-recoding of the third codon positions as starting trees. The Psiloritis haplotype group of *X. mesostena *has the same basal position as in the maximum likelihood tree obtained with the unmodified dataset and the stationary model (Figure 2A) in both resulting trees. According to the approximately unbiased test, the tree obtained using the maximum likelihood tree calculated based on the unmodified dataset as starting tree (Figure 2B) was significantly (*p *< 0.001) better than the tree obtained using the other starting tree.

Both reconstructions of the mitochondrial gene tree (Figure 2) show that the haplotypes of the morphologically delimited species *X. cretica*, *X. subvariegata*, *X. grabusana*, *X. lasithiensis*, *X. heraklea *and *X. kydonia *form monophyletic groups, but the haplotypes of the other five morphologically delimited species do not. The haplotypes of the widespread *X. mesostena *are paraphyletic with respect to all other endemic *Xerocrassa *species with the exception of *X. subvariegata *and *X. grabusana *and form two deeply separated main groups. Whereas the majority of the *X. mesostena *individuals have haplotypes that form a terminal bush-like group, most individuals living in a region southwest of the Psiloritis Mountains have mitochondrial haplotypes that form a strongly supported early branch in the mitochondrial gene tree, the 'Psiloritis haplotype group'. The haplotypes of *X. rhithymna *are paraphyletic with respect to *X. kydonia*. The haplotypes of *X. franciscoi *are nested in the major *X. mesostena *group. In the tree calculated with the stationary model (Figure 2A) most haplotypes of *X. amphiconus *form a clade that is nested in some haplotypes of *X. siderensis*, which together form the sister group of the *X. heraklea/rhithymna/kydonia *clade. The second group of *X. siderensis *haplotypes forms the sister clade of the major *X. mesostena *group. In contrast, the two haplotype clades of *X. amphiconus *and *X. siderensis *form a single clade, which is the sister group of the *X. heraklea/rhithymna/kydonia *clade, in the tree calculated with the nonstationary model (Figure 2B). In both reconstructions, one haplotype of *X. amphiconus *is nested in the major *X. mesostena *group.

### Tree and network based on AFLP data

**Figure 3:**
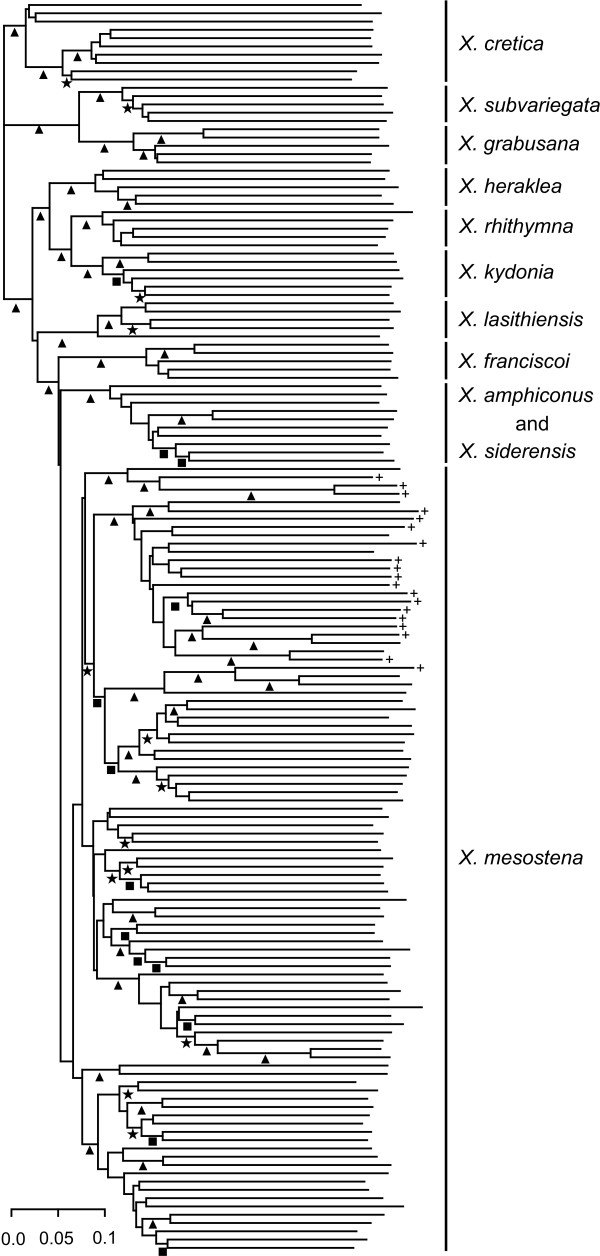
**Neighbor-joining tree based on Jaccard distances between AFLP data of Cretan *Xerocrassa***. Bootstrap support is indicated by symbols below the branches (stars = 70-80%, squares = 80-90%, triangles = 90-100%). The *X. mesostena *individuals that are characterized by a mitochondrial haplotype of the Psiloritis group are indicated by +.

**Figure 4:**
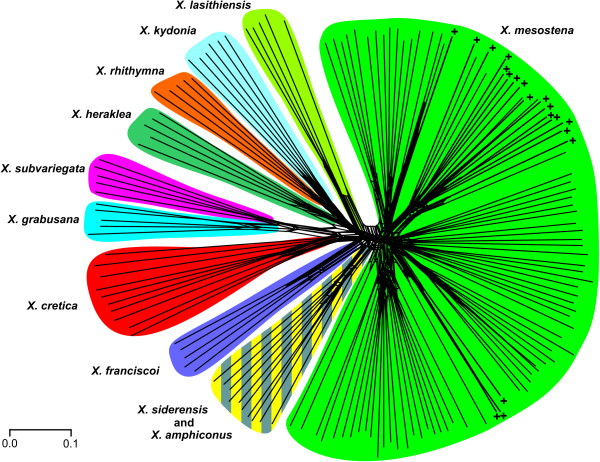
**Neighbor-net network based on Jaccard distances between AFLP data of Cretan *Xerocrassa***. The morphological classification is indicated. The *X. mesostena *individuals that are characterized by a mitochondrial haplotype of the Psiloritis group are indicated by +.

Using six primer combinations, we scored 1476 fragments of 70-322 bases length in 151 *Xerocrassa *specimens. The AFLP data can be found in Additional File 1. A neighbor-joining tree based on Jaccard distances between AFLP data of Cretan *Xerocrassa *is shown in Figure 3 and a neighbor-net is shown in Figure 4. Nine of the eleven morphologically defined species are monophyletic in the AFLP tree. This is also true for *X. rhithymna*, *X. franciscoi *and *X. mesostena*, which are nonmonophyletic in the mitochondrial gene tree. The discordances between the mitochondrial gene tree and the tree based on the AFLP data with regard to the monophyly of these species and the relationships between the species are not the result of a poor resolution of the mitochondrial gene tree according to an approximately unbiased test (*p *< 0.001). Of the morphologically defined species, only *X. amphiconus *and *X. siderensis *are polyphyletic in the mitochondrial gene tree as well as in the neighbor-joining tree and the neighbor-net based on the AFLP data. However, *X. amphiconus *and *X. siderensis *together form a separate group in the tree and the neighbor-net based on the AFLP data, whereas the haplotypes of the *X. amphiconus/siderensis *group are polyphyletic in the mitochondrial gene tree (Figure 2).

*X. mesostena *as defined by morphological characters forms a separate group in the tree (Figure 3) and the neighbor-net (Figure 4) based on AFLP markers. However, this group is divided into distinct subgroups by deep splits. These subgroups are geographically more or less separated and might be considered cryptic species. However, they are neither congruent with mitochondrial haplotype groups nor are they correlated with morphological differences. For example, one of the AFLP based subgroups is restricted to the region southwest of the Psiloritis Mountains where also the Psiloritis haplotype group occurs. Most of the *X. mesostena *individuals that are characterized by Psiloritis haplotypes are concentrated in the geographically corresponding AFLP cluster (Figs. 3 and 4). However, there are also individuals with other haplotypes in this AFLP cluster and some individuals with Psiloritis haplotypes belong to other AFLP clusters. This demonstrates that there is gene flow between the metapopulations corresponding to the AFLP clusters.

### Species delimitation based on Gaussian clustering of AFLP data

**Figure 5:**
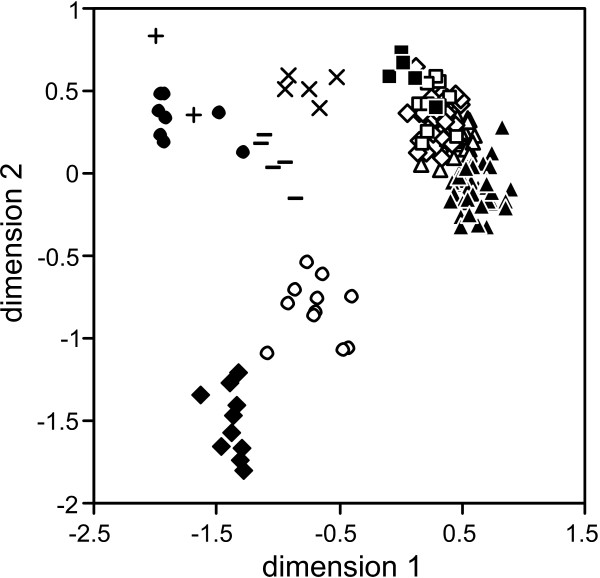
**Non-metrical multidimensional scaling of Jaccard distances between AFLP data of Cretan *Xerocrassa***. Only the first two dimensions of a four-dimensional scaling are shown. The clusters identified using Gaussian clustering correspond to the following morphologically delimited species: ▲, *X. mesostena*, partly; Δ, *X. mesostena*, partly; ◇, *X. mesostena*, partly; ◯, *X*. *kydonia *and *X*. *rhithymna*; ◆, *X. subvariegata *and *X*. *grabusana*; □, *X. amphiconus *and *X. siderensis*; ●, *X. cretica*, partly; -, *X*. *heraklea*;×, *X*. *lasithiensis*; ■, *X. franciscoi*; **+**, *X. cretica*, partly.

Gaussian clustering of the AFLP data resulted in a classification of the 151 *Xerocrassa *specimens into eleven clusters if no noise component was used. These clusters are shown in the first two dimensions of a four-dimensional non-metric multidimensional scaling of Jaccard distances (stress 12.813%; Figure 5). This partitioning differs from the morphological classification in combining the three morphologically differentiated species pairs *X. subvariegata *and *X*. *grabusana*, *X*. *kydonia *and *X*. *rhithymna*, and *X. amphiconus *and *X. siderensis *each and in splitting the widespread species *X. mesostena *into three groups and *X. cretica *into two clusters. If Gaussian clustering with a noise component was used to indicate outliers, 21 specimens were included into the noise component and seven clusters were recognized. The four clusters of the partitioning without noise that include less than eight specimens were completely included into the noise component.

### Species delimitation based on STRUCTURE analyses

**Figure 6:**
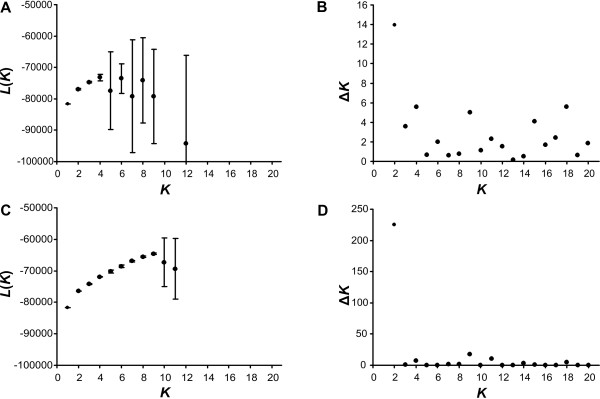
**Results of the STRUCTURE analyses of the AFLP data of Cretan *Xerocrassa *for different cluster numbers *K***. (A, B) Model without admixture. (C, D) Model with admixture. (A, C) Mean estimates of the posterior probabilities of the data for a given *K *(± SD). Only the region near the maximum is shown. Thus, posterior probabilities for higher *K *are not displayed, because their mean estimates are much smaller. (B, D) Δ*K *(following Evanno et al.[24]).

There was no distinct maximum of the mean estimates of the posterior probabilities of the data calculated with STRUCTURE for a given cluster number *K *for *K *between 1 and 20, if the model without admixture (Figure 6A) was used. The statistic Δ*K *proposed by Evanno et al. [24] to estimate the number of clusters *K *showed a maximum at *K *= 2 (Figure 6B). However, Δ*K *for *K *= 1 can not be calculated. Thus, neither the mean estimates of the posterior probabilities of the data for a given cluster number *K *nor the Δ*K *values gave a clear indication how many *Xerocrassa *species can be distinguished on Crete.

The mean estimates of the posterior probabilities of the data had a maximum at *K *= 9, if the model with admixture (Figure 6C) was used. However, the highest likelihood has been obtained in a run with *K *= 13. Δ*K *showed again a maximum at *K *= 2 (Figure 6D). In the run with *K *= 9 that had the highest likelihood the clusters correspond to *X. cretica*, *X. subvariegata *+ *X. grabusana*, *X*. *kydonia *+ *X*. *rhithymna *+ *X*. *heraklea *+ *X*. *lasithiensis*, *X*. *franciscoi*, *X. amphiconus *+ *X. siderensis *and four clusters including parts of the *X. mesostena *complex.

### Species delimitation based on a STRUCTURAMA analysis

STRUCTURAMA calculated the posterior probability that the dataset includes 3 clusters as 1.0. In the mean partition these clusters correspond to *X. cretica*, *X. subvariegata *+ *X. grabusana*, and a complex including the other eight endemic Cretan *Xerocrassa *species.

### Admixture analyses for inferring introgression

An admixture analysis with the AFLP data of *X. rhithymna *and *X. kydonia *with *K *= 2 revealed that all *X. rhithymna *individuals including the specimen with the haplotype that is sister to the *X. kydonia *haplotypes had an inferred ancestry of 99.5-99.8% in their own cluster. That means that there is no evidence for introgression of *X. kydonia *alleles into *X. rhithymna *individuals. The inferred ancestry of five *X. kydonia *individuals was also between 99.3-99.7% in their own cluster, but one individual had an inferred ancestry of only 86.5% in its own cluster and 13.5% in the *X. rhithymna *cluster.

An admixture analyses with the AFLP data of *X. franciscoi *individuals and the geographically neighbouring ten individuals of *X. mesostena *with *K *= 2 showed that the *X. franciscoi *individuals had an inferred ancestry of 93.6-99.9% in their own cluster. Nine of the ten *X. mesostena *individuals had an inferred ancestry of 99.6-99.9% in the *X. mesostena *cluster, but the one from Ano Kapetaniana 3.5 km towards Agios Ioannis, only a few hundred meters from the boundary of the distribution area of *X. franciscoi*, had an inferred ancestry of only 86.1% in the *X. mesostena *cluster and 13.9% in the *X. franciscoi *cluster. This indicates that there might be some introgression between *X. mesostena *and *X. franciscoi*.

## Discussion

### Species delimitation in a radiation

There are strong discrepancies between the mitochondrial gene tree of the radiation of the land snail genus *Xerocrassa *on Crete based on COI sequences (Figure 2) and the species classification based on morphological characters of the shell and the genitalia [21]. In the mitochondrial gene tree only six of the eleven morphologically defined *Xerocrassa *species living on Crete are monophyletic. A topology test showed that the lack of monophyly of the morphologically delimited species is not the result of a poor resolution of the mitochondrial gene tree [22]. We could also exclude the possibility that the lack of monophyly of the morphologically delimited species in the mitochondrial gene tree is an artefact resulting from systematic errors in tree reconstruction due to compositional bias by using a nonstationary model for the maximum likelihood analyses (Figure 2B).

We generated a multilocus dataset using AFLP markers to investigate whether the discrepancies between the morphologically based species classification and the mitochondrial gene tree are artefacts resulting from inadequate taxonomy or whether they can be explained by evolutionary processes. Nine of the eleven morphologically delimited Cretan *Xerocrassa *species form separate groups in the tree (Figure 3) and the neighbor-net (Figure 4) based on 1476 AFLP markers. Thus, the AFLP data corroborate the morphological species classification with the exception of the separation of *X. amphiconus *and *X. siderensis*, which together form a separate clade, but are intermingled within this clade. This species pair is also exceptional with regard to morphology and distribution. Whereas most other *Xerocrassa *species can be distinguished by characters of the genitalia, the genitalia of *X. amphiconus *and *X. siderensis *show no differences. These two species differ only in shell characters. Whereas the other endemic species have largely allopatric distribution areas, the ranges of *X. amphiconus *and *X. siderensis *broadly overlap. Nevertheless, they usually do not occur together. They tend to prefer different altitudinal zones [21], though there are several populations of each species occurring in the zone preferred by the other species. Only few individuals show intermediate shell characters indicating possible hybridization. The lack of clear genetic differentiation of the two species might indicate that the species diverged only very recently. At present, we cannot rule out the possibility that the two forms actually represent only extreme morphs of a single species.

We also applied three methods for delimiting provisional species based on dominant multilocus markers without any a priori knowledge. We compared the performance of Gaussian clustering [25,26] with two Bayesian approaches that are based on the assumption of approximate Hardy-Weinberg equilibrium within clusters, namely STRUCTURE [27,28] and STRUCTURAMA [29], in delimiting species of the Cretan *Xerocrassa *radiation.

The classification of the Cretan *Xerocrassa *specimens produced using Gaussian clustering based on the AFLP data is most similar to the morphological classification, but differs from it in combining three morphologically differentiated species pairs and in splitting the two most widespread species into two, respectively three groups. The major disadvantage of STRUCTURE was its inability to determine the number of species that can be distinguished. Compared to STRUCTURE, the advantage of STRUCTURAMA is that it directly estimates the number of clusters into which a sample can be divided. However, compared with Gaussian clustering the classification success of STRUCTURAMA was much lower. Only three provisional species were delimited. Thus, this approach failed to distinguish eight taxa that can be distinguished morphologically and form separate groups in the tree (Figure 3) and the neighbor-net (Figure 4) based on the AFLP data.

The problems in determining the appropriate number of clusters with STRUCTURE and the low resolution obtained with STRUCTURAMA confirmed former studies suggesting that with many dominant markers strategies coupling ordination and cluster analyses like Gaussian clustering become more efficient in species delimitation than Bayesian approaches that are based on the assumption of Hardy-Weinberg equilibrium within clusters [26,30]. However, none of the methods used recognized all species that can be distinguished by morphological characters and that form separate groups in the tree (Figure 3) and the neighbor-net (Figure 4) based on the AFLP data. This failure is probably the result of the low number of specimens sampled of the regionally more restricted species. With few sampled individuals separate clusters cannot be recognized and specimens of the underrepresented species are included in clusters of the most similar species, if no noise component is used in the analysis. The introduction of a noise component to include outliers in Gaussian clustering is meaningful, because it indicates problems in the data, in this case insufficient sampling of the regionally restricted species, that might remain unrecognized otherwise.

The problem in recognizing underrepresented species has important consequences for DNA-based biodiversity surveys. Because there are usually many rare species and just a few common species [31], many rare species will remain undiscovered, if all species are sampled randomly. Therefore, investing in a morphological prescreening to raise the representation of rare species in DNA-based surveys might increase the effectiveness of such surveys considerably.

The genotypic clusters determined with Gaussian clustering and the two Bayesian approaches correspond to species in some cases (e.g., *X*. *heraklea *and *X*. *lasithiensis *in the solution found with Gaussian clustering or *X. cretica *in the solution found with STRUCTURAMA), but they may also correspond to other groups like geographically isolated meta-populations (e.g., geographical subgroups of *X*. *mesostena *in the solution found with Gaussian clustering) or groups of species (e.g., *X*. *kydonia *+ *X*. *rhithymna *in the solution found with Gaussian clustering or *X. subvariegata *+ *X*. *grabusana *in the solution found with STRUCTURAMA). Thus, the determination of the status of such groups should be corroborated by a comparison with a classification based on other evidence. We compare the partitions obtained with the multilocus markers with the morphological classification that is mainly based on differences in the genitalia that may directly be involved in reproductive isolation.

### Causes of the nonmonophyly of species in the mitochondrial gene tree

The nonmonophyly of *X. mesostena*, *X. franciscoi *and *X. rhithymna *in the mitochondrial gene tree (Figure 2) are not likely explained by inadequate taxonomy, because they form separate groups in the tree (Figure 3) and the neighbor-net (Figure 4) based on AFLP markers. We analyzed the potential mechanisms resulting in the nonmonophyly of these species in the mitochondrial gene tree by applying several criteria that have been proposed to discriminate between incomplete lineage sorting and introgression. These criteria are the depth of the coalescences in the mitochondrial tree [1,18-20,32,33], the geographical occurrence of the individuals of species that are nonmonophyletic in the gene tree [1,16,18-20,34], and concordance with results of admixture analyses of nuclear multilocus markers [16,35].

The most remarkable pattern is seen in *X. mesostena*, the most widespread of the endemic species. Whereas individuals living in a region southwest of the Psiloritis Mountains have mitochondrial haplotypes that form a strongly supported early branch in the COI gene tree (Figure 2), the individuals from other regions of the island have haplotypes that form a terminal bush-like group. In the tree (Figure 3) and the neighbor-net (Figure 4) based on AFLP markers individuals with Psiloritis haplotypes are intermingled with individuals with other haplotypes indicating that there is gene flow between populations with different mitochondrial haplotype groups. There is no indication that one of the two haplotype groups found in *X. mesostena *was introduced into that species by introgression, because these haplotype groups were not found in other species, with the exception of *X. franciscoi *and one *X. amphiconus *specimen that have haplotypes that are nested in the major *X. mesostena *haplotype group. Thus, the Psiloritis haplotype group represents probably an ancestral polymorphism that was conserved in *X. mesostena*. It can be considered a paradigm for one of the causes of the high mitochondrial sequence diversity within land snail species discussed by Thomaz et al. [36], namely long-term persistence of ancient polymorphisms resulting from the strongly subdivided population structure of land snails. The population structure of land snail species often consisting of many more or less isolated populations [37-40] that sometimes can reach high densities in favourable patches of habitat in conducive to the persistence of ancestral polymorphisms, because such a population structure increases the effective population size [41]. A more detailed analysis of the phylogeographic structure within *X. mesostena *is in preparation.

The second case of nonmonophyly in the mitochondrial gene tree (Figure 2) concerns *X. rhithymna*. In the COI gene tree *X. rhithymna *is paraphyletic with respect to *X. kydonia*. The two species are sister species according to the AFLP tree. The ranges of the two species are separated by more than 40 km [21]. Thus, dispersal of *X. rhithymna *individuals to the range of *X. kydonia *and hybridization between the two species are rare events at most. This is also confirmed by an admixture analysis of the AFLP data that did not provide evidence for admixture, neither for the *X. rhithymna *individual with the haplotype that is sister to the *X. kydonia *haplotypes nor for any other *X. rhithymna *individual. A small amount of admixture in one *X. kydonia *individual is not necessarily the result of introgression, but might be due to shared ancestral polymorphisms. Thus, it is more likely that the nonmonophyly of *X. rhithymna *in the mitochondrial gene tree is the result of incomplete lineage sorting and not caused by an introgression of a *X. kydonia *haplotype into *X. rhithymna*. This is further supported by the deep coalescence of the *X. rhithymna *haplotype which is more closely related to the *X. kydonia *haplotypes than to the other *X. rhithymna *haplotypes.

The last species that is monophyletic in the AFLP tree (Figure 3), but not in the mitochondrial gene tree (Figure 2), is *X. franciscoi*. The small range of this endemic species adjoins directly on the range of *X. mesostena*. Its COI haplotypes are nested within the main COI haplotype group of *X. mesostena *and are separated from *X. mesostena *haplotypes only by shallow distances. This might indicate introgression. Actually, the STRUCTURE analysis of the AFLP data shows admixture between *X. franciscoi *and a representative of the neighbouring *X. mesostena *population in agreement with the observation of a narrow hybrid zone between the two species [21]. However, the nonmonophyly of the mitochondrial haplotypes of *X. franciscoi*, the shallow distances between them and *X. mesostena *haplotypes and the admixture of *X. franciscoi *and neighbouring *X. mesostena *can also be explained by a recent origin of *X. franciscoi *from *X. mesostena *by peripatric speciation. It is difficult to determine the relative roles of incomplete lineage sorting and hybridization in generating nonmonophyly of recently separated species in gene trees.

The mitochondrial gene tree hints to the phylogenetic origin of *X. franciscoi *from neighbouring populations of the *X. mesostena *complex, whereas the AFLP tree reflects the genetic cohesion of the individuals of each of the species caused by intraspecific gene flow and recombination that, on the other hand, obscured the details of the relations between the species. Thus, both marker types supply complementary information with regard to the phylogenetic history of the *Xerocrassa *radiation on Crete, similar to the situation concerning the radiation of the cichlid genus *Tropheus *in Lake Tanganyika [12].

The discrimination of incomplete lineage sorting of ancestral polymorphisms and introgression as causes of nonmonophyly of species in gene trees is difficult [1,16,18-20,32-35]. Our study showed that the most likely cause of nonmonophyly can be inferred at least in some cases by a combination of several criteria, namely the depth of the coalescences in the gene tree, the geographical distribution of shared genetic markers, and concordance with results of admixture analyses of nuclear multilocus markers. However, all these criteria have limitations. Randomly distributed genetic markers shared with allopatric species with limited dispersal abilities might indicate incomplete lineage sorting. However, the expectation that genetic markers that are concentrated geographically near species boundaries indicate introgression (e.g., [1,19]) is not necessarily true, because such a pattern might also be derived from a pre-existing cline in the stem species. Likewise, introgression of mitochondrial DNA cannot be completely excluded, even if there is no evidence for admixture of multilocus markers, because maternally inherited DNA like mitochondrial DNA may introgress much more rapidly through prezygotic barriers than biparentally inherited DNA [7,8]. Such shortcomings of individual criteria are ameliorated by using several criteria in an integrative approach. Gene trees of several additional genes would provide more definitive evidence for discriminating between incomplete lineage sorting and hybridization.

## Conclusion

Most cases of nonmonophyly of species in the mitochondrial gene tree of the *Xerocrassa *radiation on Crete are not caused by inappropriate morphological taxonomy, but are the result of evolutionary processes. By using nuclear multilocus data and a combination of several criteria, namely the depth of the coalescences in the gene tree, the geographical distribution of shared genetic markers, and concordance with results of admixture analyses of nuclear multilocus markers we could infer that some of these cases are probably the result of incomplete lineage sorting, whereas introgression might have been involved in other cases. Although most species are monophyletic in a tree based on the AFLP data, methods for delimiting genotypic clusters based on multilocus data alone did not recognize all these species. This is at least partly the result of an unequal representation of the species in the dataset and highlights the importance of a morphological prescreening to raise the representation of rare species in DNA-based biodiversity surveys.

## Methods

### Sampling

Snails were sampled on Crete in July/August and September/October 2004 and September/October 2005. AFLP data were determined from 150 *Xerocrassa *specimens from 124 localities on Crete covering all morphotypes and all regions of Crete (Figure 1) and one *Xerocrassa cretica *from Samos. The classification, AFLP data, locality and voucher data for the *Xerocrassa *specimens used in this study can be found in Additional File 1.

### DNA extraction

Total genomic DNA was extracted from tissue samples of the foot preserved in 100% isopropanol following the protocol proposed by Sokolov [42] with slight modifications as detailed in Sauer & Hausdorf [22].

### Mitochondrial sequences

Fragments of the cytochrome c oxidase subunit 1 (COI) gene have been previously sequenced (Sauer & Hausdorf 2009). The sequences analyzed in this paper have been deposited in GenBank under the accession numbers FJ627054-FJ627177. The used alignment is available at TreeBASE http://www.treebase.org, accession number S2413.

### AFLP

Approximately 100 ng genomic DNA were digested with 5 units EcoRI (Fermentas) at 37°C for 1 h followed by a digestion with 5 units of MseI (Fermentas) at 65°C for 1 h. 12.5 pmol of the EcoRI-adapter, 125 pmol of the MseI-adapter and 10 units of T4 DNA ligase and its buffer (GeneCraft) were added to the digestion product and incubated at 16°C for 8 h. The ligation products were diluted 1:10 with sterile ddH_2_O, and stored at -20°C.

Preselective PCR was carried out with one selective base on each primer (PA-MseI-C and PA-EcoRI-A, Table 1). 5 μl of the diluted ligation product were added to 20 μl of the preselective PCR mastermix, consisting of 15.1 μl ddH_2_O, 2.5 μl 10× PCR-buffer, 1.75 μl MgCl_2 _(50 mM), 0.25 μl dNTP (each 2 mM), 0.15 μl preselective primer mix (50 μM each), and 0.25 μl Taq-DNA polymerase (5U/μl). Preselective PCR conditions were 22 cycles of PCR (94°C for 30 s, 56°C for 30 s, 72°C for 60 s), and a final extension step at 72°C for 5 min. The quality of the preselective PCR was checked on a 1.5% agarose gel. Afterwards the products were diluted 1:20 with sterile H_2_O.

**Table 1:** Primers and fluorescent dye labels used for AFLP.

Primer	Sequence	5'- labelling
PA-EcoRI-A	5'- GAC TGC GTA CCA ATT CA -3'	None
PA-MseI-C	5'- GAT GAG TCC TGA GTA AC -3'	None
SEcoRI-CA	5'- GAC TGC GTA CCA ATT CA CA -3'	FAM
SEcoRI-CC	5'- GAC TGC GTA CCA ATT CA CC -3'	NED
SEcoRI-GG	5'- GAC TGC GTA CCA ATT CA GG -3'	HEX
SMseI-AG	5'- GAT GAG TCC TGA GTA AC AG -3'	None
SMseI-TG	5'- GAT GAG TCC TGA GTA AC TG -3'	None

Five primers with two additional bases at the 3' end (Table 1) were used for selective amplifications. Six primer combinations (SMseI/SEcoRI^DYE^) were run: AG/CA^FAM^, AG/CC^NED^, AG/GG^HEX^, TG/CA^FAM^, TG/CC^NED ^and TG/GG^HEX^. 5 μl of the diluted preselective PCR product were added to 20 μl of the selective PCR master mix consisting of 15.05 μl ddH_2_O, 2.5 μl 10× PCR-buffer, 1.75 μl MgCl_2 _(50 mM), 0.25 μl dNTP (2 mM), 0.2 μl dye primer mix (0.06 μM labelled selective EcoRI-Primer and 0.6 μM non-labelled selective MseI-Primer), and 0.25 μl Taq DNA polymerase (5U/μl). For the selective amplification a touch down PCR with a temperature decrease of 0.6°C of the annealing temperature each cycle was applied. The program starts with 94°C for 60 s, 65°C for 30 s and 72°C for 60 s followed by 13 cycles of 0.6°C decrease of annealing temperature and 1°C decrease of elongation temperature per cycle and 23 cycles with 94°C for 60 s, 56°C for 30 s and 72°C for 60 s.

1.2 μl of each of the three different primer labelled samples were mixed with 6.2 μl Hi Dye Formamid (Applied Biosystems) and 0.2 μl GS-500 ROX size standard (Applied Biosystems). The samples were denatured at 94°C for 2 min and then cooled down on ice for 4 min. The selective PCR products were electrophoretically separated using pop4-polymer (Applied Biosystems) on an ABI PRISM 3100 (Applied Biosystems) capillary sequencer.

Signal detection was performed with GeneScan version 3.1 (Applied Biosystems). Fluorescent threshold was set to 50 relative fluorescence units. The signal intensity was normalized with Genotyper version 2.5 (Applied Biosystems). Fixed fragment categories were created. A presence/absence scoring was conducted between 70 and 322 bases with a threshold set to 50 normalized units. Category spacing was set to 1 base and the category tolerance was adjusted to +/- 0.5 bases.

We exemplarily tested the reproducibility of the AFLP by repeating the steps from DNA extraction to selective PCR with one primer combination for three samples. The banding patterns were very similar in all cases. However, repeatability of fragments dropped distinctly above a fragment length larger than 322 bp. Thus, we did not score fragments above this size.

### Phylogenetic analyses

Models of sequence evolution for the maximum likelihood analyses were chosen using ModelTest version 3.7 [43] based on the Akaike Information Criterion. A partitioning of the dataset with separate models for the three codon positions was evaluated in comparison with a uniform model for the complete dataset. Maximum likelihood analyses were conducted with Treefinder [44,45]. Confidence values were computed by bootstrapping (100 replications; [46]).

We checked the homogeneity of base frequencies across taxa using the chi-square test implemented in PAUP* 4.0 beta 10 [47]. However, this test ignores correlation due to phylogenetic structure. Therefore, we also measured the probability that the base composition of two sequences is homogeneous for each pair of sequences using the matched-pairs test of symmetry as implemented in SeqVis version 1.4 [48].

To reduce compositional heterogeneity at the third codon positions we recoded these positions into 2-state categories by pooling purines (adenine and guanine: R) and pyrimidines (cytosine and thymine: Y) (RY-coding, see [49]) using the GTR2 model in Treefinder. In addition we analyzed the data set using the nonstationary model of evolution of Galtier & Gouy [50] as implemented in nhPhyML-Discrete [51], limited to 5 base content frequency categories and with 6 categories for a discrete gamma model of among-site rate variation. Trees obtained by nhPhyML-Discrete were then compared using the approximately unbiased test [52] implemented in CONSEL [53].

Jaccard distances were calculated from the AFLP data using PhylTools version 1.32 [54]. These distances were used to reconstruct neighbor-joining trees with PHYLIP version 3.66 [55] and phylogenetic networks with the neighbor-net algorithm [56] implemented in SplitsTree4 version 4.6 [57]. Confidence values for the edges of the neighbor-joining tree were computed by bootstrapping (1000 replications).

To show that the discordances between the mitochondrial gene tree and the tree based on the AFLP data are not the result of a poor resolution of the mitochondrial gene tree, we calculated the maximum-likelihood tree based on the mitochondrial sequences under the constraint that the sequences of each species form a clade (with the exception of the *X. amphiconus-siderensis *complex, which was constraint to be a single clade) as in the tree based on the AFLP data and that the relationships between the species correspond with the relationships in that tree, using the 'resolve multifurcations' option of Treefinder. Then we investigated whether this constrained tree can be rejected in comparison with the unconstrained maximum-likelihood tree by applying the approximately unbiased test [52] as implemented in Treefinder.

### Species delimitation

The delimitation of provisional species using genetic data or (presumably) genetically inherited morphological characters is based on the criterion that species are groups of organisms with similar genotypes as suggested in the genotypic cluster definition of species given by Mallet [58]. This criterion will usually be compatible with other criteria such as intrinsic reproductive isolation [59], because the lack of gene flow between reproductively isolated groups of organisms will result in an accumulation of differences due to differential adaptation and genetic drift that make these groups recognizable as different genotypic clusters. However, species in the earliest stages of speciation might differ only in a few genes that are responsible for differential adaptation or reproductive isolation. Such emerging species will hardly be recognizable using the genotypic cluster criterion.

We applied two strategies for delimiting provisional species. The first is based on a comparison of three independent datasets, namely (1) morphological data of the genitalia and the shell as summarized in the current species classification [21], (2) the structure of the mitochondrial gene tree and (3) the structure of the tree and the network based on AFLP data. Congruence between morphologically delimited groups, clades in the mitochondrial gene tree and/or in the tree or the network based on the AFLP data corroborates that such groups are evolutionary units that can be considered as provisional species.

The second strategy is to determine species boundaries based on the multilocus data without considering the morphologically based species classification or the mitochondrial sequence data. Hausdorf & Hennig [26] compared three methods for delimiting species based on dominant multilocus markers. We applied these methods to the AFLP data of the Cretan *Xerocrassa*.

Hausdorf & Hennig [26] proposed to use Gaussian clustering [25] for the determination of clusters of specimens as provisional species. We used the implementation of MCLUST [25] in the program package PRABCLUS version 2.1-1 [60] that is an add-on package for R [61]. We performed the mixture estimation for 0 to 100 clusters with as well as without a noise component for outliers. Gaussian clustering operates on a dataset where the cases are defined by variables of metric scale. Therefore we performed a non-metric multidimensional scaling [62] using four dimensions and Jaccard distances as recommended by Link et al. [63] for dominant genetic markers.

Shaffer & Thomson [9] proposed to use STRUCTURE [27,28] to delimit the lower bound of potential species. STRUCTURE is a model-based clustering method using multilocus data to infer population structure and assign individuals to populations under the assumption of Hardy-Weinberg equilibrium within populations. We used STRUCTURE version 2.3.1 with the model without admixture as well as with the model with admixture. 10 runs with 100,000 iterations after a burn-in of 10,000 iterations were carried out in order to quantify the amount of variation of the posterior probabilities of the data for each cluster number *K *for *K *between 1 and 20. We used the mean estimates of the posterior probabilities of the data for a given cluster number *L*(*K*) and the statistic *ΔK *proposed by Evanno et al. [24] to estimate the number of clusters.

We also applied the program STRUCTURAMA [29] that is also a Bayesian approach designed for inferring population structure by clustering individuals such that Hardy-Weinberg equilibrium is maximized within clusters. However, in contrast to STRUCTURE, the number of clusters and assignment of individuals to clusters can both be considered random variables that follow a Dirichlet process prior. Thus, this method can directly estimate the number of clusters in which a sample can be divided. We allowed the number of populations to be a random variable with a Dirichlet process prior, run the Markov chain Monte Carlo analysis for 1,000,000 cycles, sampled every 100th cycle, and discarded the first 4,000 samples as burn-in.

### Admixture analysis for inferring introgression

We used admixture analyses of the AFLP data with STRUCTURE also to investigate whether cases of nonmonophyly in the mitochondrial gene tree can be ascribed to introgression. For that purpose the species were analysed pairwise with *K *= 2. We carried out 5 separate runs with 100,000 iterations after a burn-in of 10,000 iterations and analysed the run with the highest likelihood.

## Authors' contributions

BH designed the study. Both authors sampled specimens. JS carried out the molecular genetic studies. Both authors were involved in data analyses. The manuscript was drafted by BH with contributions by JS. Both authors read and approved the final version.

## Supplementary Material

Additional file 1 **Classification, AFLP, locality and voucher data for the *Xerocrassa *specimens used in this study**. ZMH, Zoological Museum Hamburg. Click here for file
